# Dramatic Response to Lidocaine Infusion Therapy for Chronic Generalized Pain Secondary to Multiple Large Keloids

**DOI:** 10.7759/cureus.96109

**Published:** 2025-11-04

**Authors:** Seung J Lee, Samuel Maccormick, Kanchana Gattu

**Affiliations:** 1 Anesthesiology, University of Maryland School of Medicine, Baltimore, USA; 2 Anesthesiology, University of Maryland Medical Center, Baltimore, USA

**Keywords:** chronic pain management, intravenous lidocaine, keloid pain, neuropathic pain, post-infectious keloid

## Abstract

Keloids are benign fibroproliferative lesions that may develop after trauma or infection, including chickenpox, and can cause chronic pain and functional impairment. Although lidocaine infusions have demonstrated efficacy in treating various neuropathic pain syndromes, their use for keloid-associated pain has not been well described. We present a case of a 45-year-old female with chronic, intractable generalized body pain secondary to multiple large keloids, developed following a chickenpox infection at the age of seven. Conventional treatments, including surgical excision with intralesional corticosteroids, duloxetine, meloxicam, amitriptyline, and acupuncture, provided only transient relief. Moderate pain relief was achieved with pregabalin, nortriptyline, topical lidocaine, and medical marijuana, resulting in a visual analog scale (VAS) score of 8/10. The subsequent addition of intravenous (IV) lidocaine infusions (5 mg/kg administered over one hour) produced significant pain reduction (VAS 3/10) and notable improvements in quality of life and functional capacity, enabling her to maintain employment. The interval between infusions was later extended to every four months while maintaining pain relief, without neuroexcitatory or cardiac complications. This case suggests that periodic IV lidocaine infusions may offer a safe and effective therapeutic option for sustained analgesia in patients with chronic keloid-related pain.

## Introduction

Keloids are benign fibroproliferative growths that extend beyond the boundaries of the original wound and result from an abnormal wound-healing process [1]. They are characterized by excessive collagen deposition and persistent inflammation, often leading to disfigurement, pruritus, and pain. Although keloids can arise from a variety of causes, including trauma, burns, or surgical incisions, post-infectious keloid formation, such as that following a chickenpox infection, has also been reported [2]. These lesions can cause substantial psychosocial distress and chronic pain, particularly when widespread or located in areas of motion and clothing friction.

Conventional treatments for keloids include intralesional corticosteroid injections, surgical excision, cryotherapy, pressure therapy, silicone gel application, and radiation [3]. Pharmacologic treatments such as antihistamines, antidepressants, and anticonvulsants may provide partial symptomatic relief but often fail to achieve sustained pain control. In refractory cases, the chronic keloid pain can be neuropathic in nature and may not respond adequately to traditional analgesics, requiring alternative systemic or interventional approaches [4].

Intravenous (IV) lidocaine infusion therapy has been demonstrated to be effective in managing several types of chronic neuropathic pain syndromes, including complex regional pain syndrome, fibromyalgia, and postherpetic neuralgia [5]. Lidocaine, a sodium channel blocker, exerts analgesic and antihyperalgesic effects by inhibiting ectopic neuronal firing and modulating central sensitization. However, the use of IV lidocaine for keloid-related pain has not been widely reported in the literature.

This case report describes a patient with chronic, intractable, generalized body pain secondary to multiple widespread keloids that developed after a childhood chickenpox infection. The case highlights the significant and sustained pain relief achieved through the adjunctive use of IV lidocaine infusions in combination with multimodal pharmacotherapy.

## Case presentation

**Figure 1:**
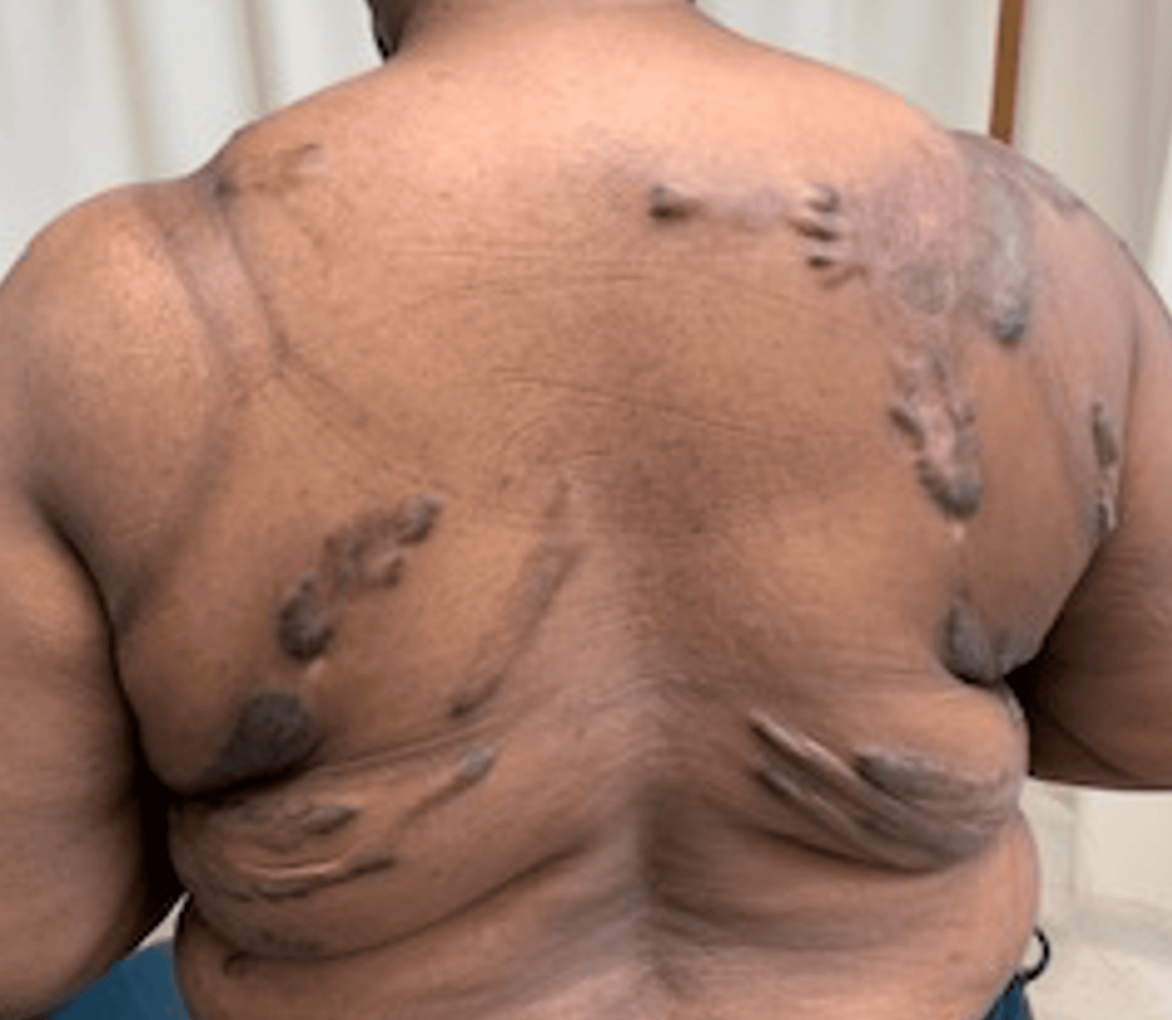
Keloid scar on the body

**Figure 2:**
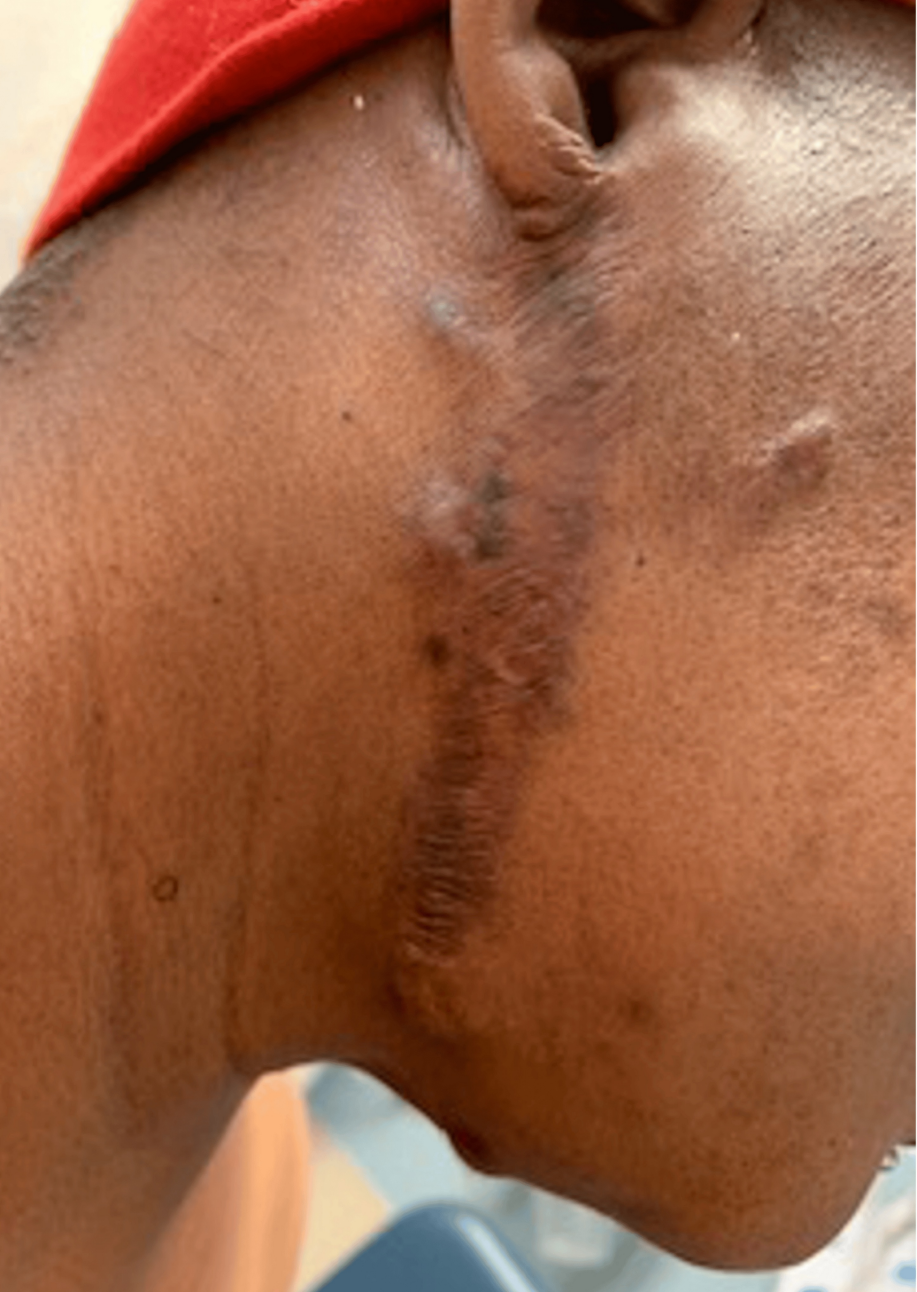
Keloid scar on the neck

**Figure 3:**
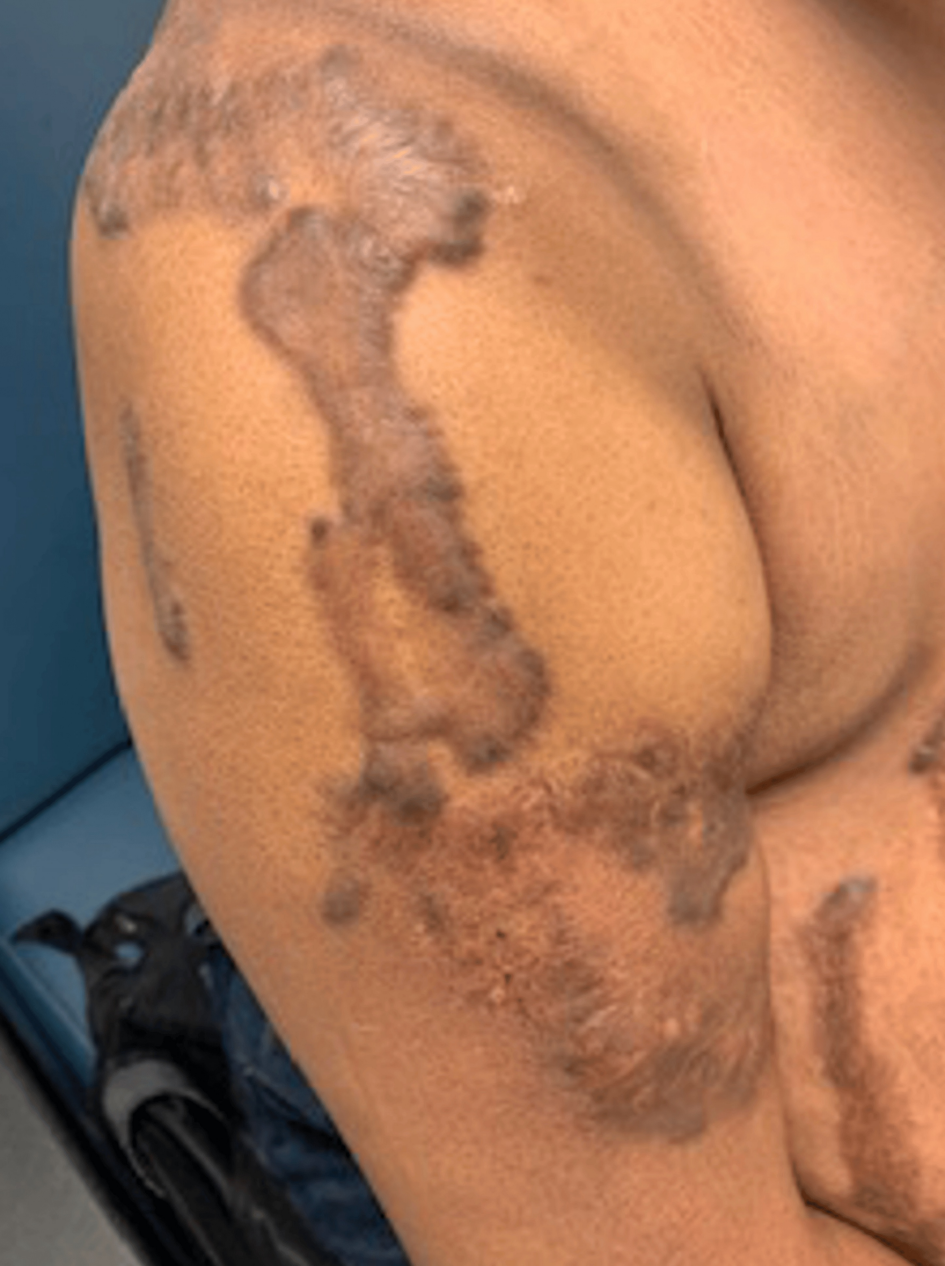
Keloid scar on the arm

A 45-year-old female with a history of chronic intractable generalized body pain secondary to multiple large keloids presented to our outpatient academic pain management clinic. Her keloids originated from a chickenpox infection sustained at the age of seven, which resulted in widespread scarring involving her neck, chest, back, and extremities (Figures 1-3). She reported persistent, burning, and throbbing pain localized to and surrounding the keloid sites, significantly impairing her mobility, sleep, and quality of life. Her baseline pain intensity was 8/10 on the visual analog scale (VAS).

The patient had previously undergone multiple conventional treatment modalities, including surgical excision of the keloids with intralesional corticosteroid injections, duloxetine, meloxicam, amitriptyline, and acupuncture, all of which provided only short-lasting or minimal benefit. She had also attempted non-pharmacologic approaches such as yoga, meditation, exercise, and dietary modification, with negligible improvement. Prior to the lidocaine infusion therapy, she had achieved partial and temporary pain relief with a combination regimen of pregabalin, nortriptyline, topical lidocaine ointment, and medical marijuana.

Given the neuropathic characteristics of her pain and its resistance to multimodal conservative therapy, IV lidocaine infusion therapy was initiated. The patient received 5 mg/kg of lidocaine administered over one hour every two months under continuous cardiac and neurological monitoring. After the first infusion, she reported a marked reduction in pain (VAS 3/10) and improvement in daily function, mood, and sleep quality. Following several sessions, the duration of analgesic benefit extended up to four months, at which point the infusion interval was lengthened accordingly. She experienced no adverse effects, including neuroexcitatory symptoms or cardiac conduction abnormalities, throughout her treatment course.

The patient has continued to maintain meaningful pain control and functional improvement with periodic IV lidocaine infusions as part of a comprehensive, multimodal pain management plan for the last five years.

## Discussion

Keloids are benign fibroproliferative lesions that result from an abnormal wound-healing response characterized by excessive collagen deposition and persistent inflammation [1]. They often extend beyond the boundaries of the original wound and can lead not only to cosmetic concerns but also to chronic pain, pruritus, and functional impairment. Pain associated with keloids may result from the entrapment or irritation of peripheral nerve fibers within dense fibrotic tissue, as well as the release of inflammatory mediators that lead to peripheral sensitization [6]. Refractory pain may exhibit neuropathic characteristics, presenting as burning, shooting, or electric-like sensations.

Conventional treatment modalities for keloid pain focus primarily on addressing scar tissue formation and inflammation through excision, corticosteroid injection, silicone sheeting, cryotherapy, or radiation [3]. However, these approaches often fail to alleviate neuropathic components of pain and may even exacerbate symptoms through recurrent tissue trauma or nerve irritation. Pharmacologic management with antidepressants, anticonvulsants, and topical agents can provide partial relief but may be limited by side effects or diminishing efficacy over time. Consequently, there is a growing interest in exploring alternative systemic therapies that target abnormal pain signaling pathways more directly [4].

IV lidocaine has gained increasing attention as a therapeutic option for refractory neuropathic pain syndromes, including complex regional pain syndrome, fibromyalgia, and postherpetic neuralgia [5]. Lidocaine acts primarily through voltage-gated sodium channel blockade, inhibiting ectopic discharges in damaged or sensitized nerves and modulating both peripheral and central pain transmission. Additionally, it exhibits anti-inflammatory properties by suppressing the release of proinflammatory cytokines and reducing microglial activation within the central nervous system. These mechanisms may collectively contribute to both immediate and sustained analgesic effects following systemic administration [7].

Our patient’s response to periodic IV lidocaine infusions suggests that this treatment modality may offer durable pain relief in cases of chronic keloid-related pain refractory to conventional therapies. The observed prolongation of analgesic duration, from two to four months between infusions, may reflect neuroplastic modulation or prolonged suppression of abnormal nociceptive signaling. Importantly, the treatment was well tolerated, with no evidence of neuroexcitatory or cardiac conduction abnormalities, underscoring the safety of the dosing regimen when administered under appropriate monitoring.

While limited data exist on the use of IV lidocaine for keloid pain, its efficacy in other chronic neuropathic pain conditions supports its potential therapeutic role. Further research, including controlled trials and mechanistic studies, is warranted to elucidate the optimal dosing protocols, frequency, and predictors of response in this unique patient population. Understanding the intersection of fibrosis, inflammation, and neuropathic pain in keloid pathology may also open new avenues for targeted multimodal treatment strategies.

## Conclusions

This case highlights the potential role of IV lidocaine infusion therapy as a safe and effective treatment for chronic pain secondary to widespread keloids refractory to conventional management. The sustained pain relief and functional improvement observed suggest that periodic IV lidocaine infusions may provide long-term benefit in select patients. Further studies are needed to explore the mechanisms, optimal dosing intervals, and long-term safety of lidocaine infusion in the management of keloid-associated pain.
